# Response to the Letter to the Editor regarding ‘The factor structure of complex posttraumatic stress disorder in traumatized refugees’

**DOI:** 10.1080/20008198.2017.1308200

**Published:** 2017-04-03

**Authors:** Angela Nickerson, Marylene Cloitre, Richard A Bryant, Ulrich Schnyder, Naser Morina, Matthis Schick

**Affiliations:** ^a^ School of Psychology, UNSW Australia, Sydney, NSW, Australia; ^b^ New York University School of Medicine, New York University, New York, NY, USA; ^c^ Department of Psychiatry and Psychotherapy, Zurich University Hospital, Zurich, Switzerland

We are writing in response to the Letter to the Editor by De Jongh and colleagues regarding our article ‘The factor structure of complex posttraumatic stress disorder in traumatized refugees’ (Nickerson et al., 2016). The authors raise several queries about the methodology employed in our study, asserting that ‘there were a number of flaws that we consider as potential threats to both the internal and external validity of the study results and, therefore, compromise confidence in the conclusions of the authors’. We appreciate the opportunity to respond to this Letter, and to correct several errors made in the critique of this and related studies.

The first methodological criticism raised by De Jongh and colleagues relates to the assessment of traumatic events the sample may have experienced and of the complex PTSD (CPTSD) diagnosis. They queried whether participants in the current study met criteria for CPTSD, arguing that it was not possible to determine whether participants had experienced an event that was sufficiently traumatic to qualify for the diagnoses of CPTSD and PTSD without conducting clinical interviews. As reported in the manuscript, over 90% of the sample reported having experienced or directly witnessed torture on a self-report measure of trauma exposure. The remaining participants reported having been exposed to a mean of 8.67 (standard deviation = 3.97) types of traumatic events, with a range of 4–15 types of traumas. These events included physical assault, sexual assault and witnessing the murder of family and friends. Thus, all participants in this study had been exposed to one or more events that would meet the definition of traumatic according to the DSM-IV criteria. De Jongh and colleagues have questioned the validity of determining diagnostic status in the absence of clinical interviews. We disagree that this represents ‘improper assessment procedure’. As reported in the article (and as noted by De Jongh and colleagues), we used self-report measures to assess *probable* diagnostic status. We agree with the authors that future research investigating CPTSD in traumatized refugees could benefit from implementing clinical interviews to determine diagnostic status.

De Jongh and colleagues also queried whether CPTSD symptoms in the current sample may have been associated with non-trauma-related stressors, such as those experienced in displacement. We agree with the authors that the significant challenges experienced by refugees in the post-migration environment may exacerbate symptoms of PTSD and CPTSD. In fact, there is a large body of evidence linking post-migration stressors to symptoms of PTSD and other mental health difficulties (Li, Liddell, & Nickerson, 2016; Porter & Haslam, 2005). It is thus likely that symptoms of both PTSD and CPTSD (e.g. feelings of worthlessness, feeling cut-off from others) arising from trauma exposure will be heightened in the post-migration environment. For example, many refugees are separated from family members who remain in peril in the country of origin which might directly contribute to symptoms like nightmares, hypervigilance and feelings of guilt. The likelihood that these symptoms may be heightened by post-migration stressors should not be interpreted as evidence that the CPTSD construct is not applicable to this group; on the contrary, it provides a strong rationale for examining both CPTSD and PTSD in refugee groups.

In addition, the authors also suggested that symptom endorsement may have arisen from comorbidity given the use of items from measures designed to assess other psychological constructs (e.g. depression) to index CPTSD. While it is indeed the case that there are similarities between items measured in the current study and symptoms of other disorders such as depression, findings from the current study indicated that the items measuring disturbances in self-organisation loaded onto the affect regulation, self-concept and interpersonal relationships factors as hypothesized. This provided preliminary evidence that the identified symptoms form part of a cohesive construct in traumatized refugees. Such symptoms (e.g. low self-worth) are currently typically recognized by the addition of multiple comorbid disorders. This can lead to the excessive pathologizing of the patient as every new diagnosed disorder brings with it additional peripheral symptoms and creates a challenge to treatment formulation which, to date, remains unresolved. This is reflected in the finding that the majority of trauma-exposed individuals with PTSD have three or more co-morbid diagnoses (Kessler, Sonnega, Bromet, Hughes, & Nelson, 1995). We propose that CPTSD presents a coherent and streamlined profile of the critical symptoms that frequently occur following exposure to trauma, particularly chronic forms. Future research is needed to examine the potential benefits of the CPTSD diagnosis as an efficient and precise constellation of correlated symptoms that present together, persist across time together and, under effective treatment, resolve together.

From a theoretical perspective, De Jongh and colleagues queried the relevance of the CPTSD construct to refugees, stating that ‘the authors mention a series of studies (De Jong, Komproe, Spinazzola, Van der Kolk, & Van Ommeren, 2005; Palic & Elklit, 2014; Morina & Ford, 2008) to substantiate their argument that CPTSD may be particularly relevant to refugee groups. Unfortunately, they failed to mention that, in the cited studies, CPTSD prevalence was found to be low when assessed by using diagnostic interviews’. As stated in the article, the cited studies were undertaken based on the rationale that CPTSD and related constructs may be especially relevant to individuals from non-western countries who have been exposed to persecution, mass trauma and torture (de Jong, Komproe, Spinazzola, van der Kolk, & van Ommeren, 2005; Cloitre et al., 2014; Morina & Ford, 2008). It is important to note that these studies investigated the prevalence of Disorders of Extreme Stress Not Otherwise Specified, which is conceptually related to CPTSD but assesses a much broader range of symptoms. The symptom clusters encompassed in this diagnosis include those that are characteristic of CPTSD (disruptions in affect and impulses, self-perception, relationships with others), as well as those that are outside the scope of the diagnosis proposed for ICD-11 and measured in this study (disruptions in attention and consciousness, somatization and disruptions in systems of meaning). The prevalence of this diagnosis was indeed found to be low to moderate in the cited studies, but the prevalence of symptoms characteristic of CPTSD was relatively higher. For example, in the study conducted by de Jong et al., rates of DESNOS ranged from 2% (Ethiopia) to 13% (Algeria), however endorsement of individual symptom clusters were as follows: alterations in affect and impulse regulation ranged from 13 to 35%, alterations in self-perception ranged from 15 to 31% and alterations in relations with others ranged from 25 to 66% (de Jong et al., 2005). In the study conducted by Morina and Ford, 2% of the sample met criteria for DESNOS, however 10% showed affect dysregulation, 15% altered self-perception and 34% altered relationships with others (Morina & Ford, 2008). In the study conducted by Palic and Elklit, 34% of the sample met criteria for full DESNOS, while 23% met criteria for five out of the six symptom clusters (Palic & Elklit, 2014). This led the authors in these studies to conclude that, while the full DESNOS construct may not be appropriate for these groups, aspects of the disorder are highly relevant and further investigation is thus required.

De Jongh and colleagues queried the statistical validity of the findings in the current study. First, we reject as flippant the assertion by De Jongh and colleagues that the testing of two models when there are an ‘infinite number of models’ that could be tested represents a fundamental flaw in the analyses. It is incorrect to state that there are an infinite number of models that can be tested. CFA is a statistical procedure that allows for the evaluation of the validity of theoretically-informed explanations for the clustering of data. It is not a statistical technique appropriate for open-ended testing of models, as EFA might be. The two models that were tested in this study were chosen to directly investigate the question of whether DSO and PTSD were distinct but related constructs. This study posed a specific question regarding the nature of the second-order structure of CPTSD symptoms and the two models tested were appropriate and sufficient in that they evaluated a priori, theoretically generated proposals concerning plausible structures of the data.

De Jongh and colleagues also erroneously stated that our finding that the two-factor model fitted the data better than the one-factor model was incorrect. In this study we implemented recommended indices for comparing model fit, including chi-square, RMSEA, CFI, TLI, AIC and SS-BIC (Hu & Bentler, 1999). As noted by the authors, the chi-square value was significant for the one-factor model and not the two-factor model. This indicates that the two-factor model better fits the data. Further, all other indices used to evaluate the model fit (RMSEA, CFI, TLI, AIC and SS-BIC) indicated that the two-factor model evidenced superior fit. It is notable that this was even the case for model comparison indices (i.e. SS-BIC and AIC) which include a penalty for increasingly complex models. De Jongh and colleagues’ assertion that a correlation of 0.84 between PTSD and DSO indicates that these factors ‘virtually measure the same construct’ is also incorrect. A correlation of 0.84 between PTSD and DSO indicates that the two factors share 70.56% of the variance in common. If these two factors did indeed measure the same construct, then the higher-order model with one factor would have demonstrated better fit, but it did not. In addition, it is likely that the two-factor higher-order model would have either (1) evidenced unsatisfactory model fit (because the implied correlation was not reflective of the nature of the sample covariance matrix), or (2) the factorial solution would not have converged in a meaningful manner that allowed for the interpretation of model fit. Instead, the two-factor model yielded superior fit to the one-factor model on every index including those that favoured more parsimonious models. Overall, these results were consistent with the findings of three other publications that have systematically tested a range of models and have found that the two-factor, higher-order model best fit the data (Hyland et al., 2016; Karatzias et al., 2016; Shevlin et al., 2017).

Overall, the assertion that our findings ‘do not lend much credence to the existence of two clear and distinguishable symptom profiles’ is incorrect. Our study found support for the distinction between PTSD and CPTSD as proposed for the ICD-11. Contrary to the assertion of De Jongh and colleagues, this points to the existence of two patient groups: one whose symptoms map onto classic PTSD conceptualizations, and another who also experience additional disruptions to affect regulation, interpersonal relationships and self-concept. Accordingly, it may be the case that different interventions are warranted for these groups. There are many possible interventions for more complex presentations of PTSD. We disagree with the dismissal by De Jongh about the potential value of skills interventions, such as Skills Training in Affective and Interpersonal Regulation (STAIR). In a well-designed study, the comparison of STAIR preceding exposure (STAIR+Ex) relative to Supportive Counseling preceding exposure (SC+Ex) indicated that the first condition resulted in fewer dropouts, less symptom exacerbation during exposure, superior benefits and less relapse in follow-up (Cloitre et al., 2010). De Jong and colleagues dismiss the trial with the claim that the use of supportive counselling adversely affected the use of exposure (De Jongh et al., 2016). In fact, the dropout rates in SC+Ex condition (39.4%) were quite consistent with the results of direct-to-exposure studies that included chronically traumatized women: 38% female veterans (Schnurr et al., 2007); 38.7% female veterans (Eftekhari et al., 2013). SC did not increase PTSD symptoms preceding exposure but rather provided significant reductions, suggesting that it may benefit rather than exacerbate patient problems. It was simply the case that STAIR was superior to SC in providing symptom reduction. More research is needed to assess the potential benefits of treatments with sequentially organized interventions, and to determine what types of interventions are most effective and for whom. Meta-analytic data from PTSD clinical trials tell us that nearly half of all patients do not remit from the disorder (Bisson, Roberts, Andrew, Cooper, & Lewis, 2013). This state of affairs indicates the need to identify patients for whom current treatments are not optimal or not effective and investigate alternative treatment formulations. An open mind in exploring options is essential to the conduct of good science and improving outcomes for various trauma-exposed populations.

We thank De Jong and colleagues for drawing attention to the importance of investigating complex psychological responses to refugee trauma. In the context of an increasing body of literature supporting the validity of CPTSD across populations (Cloitre, Garvert, Brewin, Bryant, & Maercker, 2013; Cloitre et al., 2014; Elklit, Hyland, & Shevlin, 2014; Karatzias et al., 2016; Murphy, Elklit, Dokkedahl, & Shevlin, 2016; Palic et al., 2016; Perkonigg et al., 2016), this initial study points to the need for further research into understanding how psychological responses to prolonged interpersonal traumatic events may be manifested in refugee groups. While our understanding as to the complexity of these traumatic stress responses in refugees is in its infancy, this first study highlights the potential utility of the CPTSD diagnosis in conceptualizing these reactions and points to treatment pathways that may be beneficial in these groups. Given there are currently over 65 million people forcibly displaced worldwide (UNHCR, 2016), it is critical that we build a strong evidence base if we are to advance knowledge regarding the mental health of refugees. This evidence will directly inform the development and implementation of evidence-based interventions to alleviate the psychological impact of refugee trauma and torture.

## References

[CIT0001] Bisson J. I., Roberts N. P., Andrew M., Cooper R., Lewis C. (2013). Psychological therapies for chronic post-traumatic stress disorder (PTSD) in adults. *Cochrane Database of Systematic Reviews (Online)*.

[CIT0002] Cloitre M., Garvert D. W., Brewin C. R., Bryant R. A., Maercker A. (2013). Evidence for proposed ICD-11 PTSD and complex PTSD: A latent profile analysis. *European Journal of Psychotraumatology*.

[CIT0003] Cloitre M., Garvert D. W., Weiss B., Carlson E. B., Bryant R. A. (2014). Distinguishing PTSD, Complex PTSD, and Borderline Personality Disorder: A latent class analysis. *European Journal of Psychotraumatology*.

[CIT0004] Cloitre M., Stovall-McClough K. C., Nooner K., Zorbas P., Cherry S., Jackson C. L., Petkova E. (2010). Treatment for PTSD related to childhood abuse: A randomized controlled trial. *The American Journal of Psychiatry*.

[CIT0005] de Jong J. T., Komproe I. H., Spinazzola J., van der Kolk B. A., van Ommeren M. H. (2005). DESNOS in three postconflict settings: Assessing cross-cultural construct equivalence. *Journal of Traumatic Stress*.

[CIT0006] De Jongh A., Resick P. A., Zoellner L. A., van Minnen A., Lee C. W., Monson C. M., Bicanic I. A. (2016). Critical analysis of the current treatment guidelines for complex Ptsd in adults. *Depression and Anxiety*.

[CIT0007] Eftekhari A., Ruzek J. I., Crowley J. J., Rosen C. S., Greenbaum M. A., Karlin B. E. (2013). Effectiveness of national implementation of prolonged exposure therapy in Veterans Affairs care. *JAMA Psychiatry*.

[CIT0008] Elklit A., Hyland P., Shevlin M. (2014). Evidence of symptom profiles consistent with posttraumatic stress disorder and complex posttraumatic stress disorder in different trauma samples. *European Journal of Psychotraumatology*.

[CIT0009] Hu L., Bentler P. M. (1999). Cutoff criteria for fit indexes in covariance structure analysis: Conventional criteria versus new alternatives. *Structural Equation Modeling*.

[CIT0010] Hyland P., Shevlin M., Elklit A., Murphy J., Vallieres F., Garvert D. W., Cloitre M. (2016). An Assessment of the Construct Validity of the ICD-11 Proposal for Complex Posttraumatic Stress Disorder. *Psychological Trauma: Theory, Research, Practice and Policy*.

[CIT0011] Karatzias T., Shevlin M., Fyvie C., Hyland P., Efthymiadou E., Wilson D., Cloitre M. (2016). An initial psychometric assessment of an ICD-11 based measure of PTSD and complex PTSD (ICD-TQ): Evidence of construct validity. *Journal of Anxiety Disorders*.

[CIT0012] Kessler R. C., Sonnega A., Bromet E., Hughes M., Nelson C. B. (1995). Posttraumatic stress disorder in the National Comorbidity Survey. *Archives of General Psychiatry*.

[CIT0013] Li S. S., Liddell B. J., Nickerson A. (2016). The relationship between post-migration stress and psychological disorders in refugees and asylum seekers. *Current Psychiatry Reports*.

[CIT0014] Morina N., Ford J. D. (2008). Complex sequelae of psychological trauma among Kosovar civilian war victims. *The International Journal of Social Psychiatry*.

[CIT0015] Murphy S., Elklit A., Dokkedahl S., Shevlin M. (2016). Testing the validity of the proposed ICD-11 PTSD and complex PTSD criteria using a sample from Northern Uganda. *European Journal of Psychotraumatology*.

[CIT0016] Nickerson A., Cloitre M., Bryant R. A., Schnyder U., Morina N., Schick M. (2016). The factor structure of complex posttraumatic stress disorder in traumatized refugees. *European Journal of Psychotraumatology*.

[CIT0017] Palić S., Elklit A. (2014). Personality dysfunction and complex posttraumatic stress disorder among chronically traumatized Bosnian refugees. *The Journal of Nervous and Mental Disease*.

[CIT0018] Palic S., Zerach G., Shevlin M., Zeligman Z., Elklit A., Solomon Z. (2016). Evidence of complex posttraumatic stress disorder (CPTSD) across populations with prolonged trauma of varying interpersonal intensity and ages of exposure. *Psychiatry Research*.

[CIT0019] Perkonigg A., Höfler M., Cloitre M., Wittchen H. U., Trautmann S., Maercker A. (2016). Evidence for two different ICD-11 posttraumatic stress disorders in a community sample of adolescents and young adults. *European Archives of Psychiatry and Clinical Neuroscience*.

[CIT0020] Porter M., Haslam N. (2005). Predisplacement and postdisplacement factors associated with mental health of refugees and internally displaced persons: A meta-analysis. *Journal of the American Medical Association*.

[CIT0021] Schnurr P. P., Friedman M. J., Engel C. C., Foa E. B., Shea M. T., Chow B. K., Bernardy N. (2007). Cognitive behavioral therapy for posttraumatic stress disorder in women: A randomized controlled trial. *JAMA*.

[CIT0022] Shevlin M., Hyland P., Karatzias T., Fyvie C., Roberts N., Bisson J. I., Cloitre M. (2017). Alternative models of disorders of traumatic stress based on the new ICD-11 proposals. *Acta Psychiatrica Scandinavica*.

[CIT0023] UNHCR (2016). 2016 global trends report. http://www.humansecuritybrief.info/index.html.

